# Genome of the Haloarchaeon *Natronomonas moolapensis*, a Neutrophilic Member of a Previously Haloalkaliphilic Genus

**DOI:** 10.1128/genomeA.00095-13

**Published:** 2013-03-21

**Authors:** Mike L. Dyall-Smith, Friedhelm Pfeiffer, Tanja Oberwinkler, Kathrin Klee, Markus Rampp, Peter Palm, Karin Gross, Stephan C. Schuster, Dieter Oesterhelt

**Affiliations:** a School of Biomedical Sciences, Charles Sturt University, Wagga Wagga, NSW, Australia; b Department of Membrane Biochemistry, Max Planck Institute of Biochemistry, Martinsried, Germany; c Computing Center (RZG) of the Max Planck Society, Max Planck Institute of Plasma Physics, Garching, Germany; d Center for Comparative Genomics and Bioinformatics, Center for Infectious Disease Dynamics, Penn State University, University Park, Pennsylvania, USA; e Singapore Centre on Environmental Life Sciences Engineering, and School of Biological Sciences, Nanyang Technological University, Singapore

## Abstract

The genus *Natronomonas* contains two species, one haloalkaliphile (*N. pharaonis*) and one neutrophile (*N. moolapensis*). Here, we report the genome sequence of *N. moolapensis* strain 8.8.11. The overall genome properties are similar for the two species. Only the neutrophile contains bacteriorhodopsin and a membrane glycolipid.

## GENOME ANNOUNCEMENT

The two species currently assigned to the genus *Natronomonas* are *N. pharaonis*, a haloalkaliphile isolated from the Wadi Natrun in Egypt (1) (which was previously sequenced) (2), and *N. moolapensis*, a neutrophile that grows optimally at a pH of 7 to 7.5 and was recovered from a solar saltern in southeastern Australia (3). The genome of *N. moolapensis* strain 8.8.11 (JCM14361^T^ = DSM18674^T^) was sequenced by the same strategy as described recently (4). Briefly, contigs from standard 454 whole-genome sequencing (171.4 Mb of raw data, assembled by Newbler into 118 contigs with 2.85 Mb and average coverage of 60.1-fold) were ordered by end-paired Sanger sequencing of cosmid clones (2,857 cosmid end sequences). Adjacent contigs were spanned by PCRs to close the genome. The final assembly was prepared using the Phred-Phrap-Consed package (5). The genome was annotated according to *N. pharaonis* and other haloarchaeal genomes maintained at a high annotation quality level within the HaloLex framework (6). Additional bioinformatic tools were used as described previously (4).

There is a single chromosome of 2.9 Mb with a G+C content of 64.5%, a single rRNA operon, 46 tRNA genes, and no plasmids. The sequence CTAG was strongly avoided. A partial tRNA-Glu gene is associated with a likely prophage (nucleotides [nt] 1501283 to 1545787).

The genome codes for 2,846 proteins, of which 1,930 (67.8%) have a bidirectional best BLAST match in *N. pharaonis*, with an average of 70% sequence identity.

The strain is motile and cells display intracellular gas vesicles (3), consistent with the presence of genes for flagella (e.g., *flg1* to *flg3*), signal transduction (e.g., *cheA, cheY*), sensory rhodopsin, and a gas vesicle cluster. By microscopy, intracellular granules were observed in pyruvate-grown cells, consistent with the presence of polyhydroxyalkanoate biosynthesis genes. No Cas/CRISPR genes were found. The cell membrane of *N. moolapensis* contains a prominent glycolipid, consistent with the presence of bacteriorhodopsin, as described previously for *Halobacterium* (7, 8). By comparison, *N. pharaonis* and other haloalkaliphilic archaea have neither *bop* nor membrane glycolipids. Studies of haloarchaeal diversity in China and Iran revealed 16S rRNA sequences with 99% identity to *N. moolapensis* (accession no. GQ282622 and HQ425158), indicating a wide distribution of this species.

Several enzymes are found in *N. moolapensis* but not in *N. pharaonis*. Among these is a glycerol utilization cluster (glycerol kinase and heterotrimeric glycerol-3-phosphate dehydrogenase), explaining why *N. moolapensis* but not *N. pharaonis* can grow on glycerol. In contrast to *N. pharaonis*, *N. moolapensis* has most enzymes of the Entner-Doudoroff pathway. Despite this feature, however, the sequenced type strain (8.8.11) was not found to grow on glucose, while this is possible for a different strain of *N. moolapensis*, strain 4.03.5 (3). A *puc* gene cluster for urate-to- allantoin conversion occurs in *N. moolapensis* and only three other sequenced haloarchaeal genomes: those of *Haloferax volcanii*, *Halalkalicoccus jeotgali*, and *Haloquadratum walsbyi*. In the latter organism, it is found in the type strain C23, isolated from the same saltern as *N. moolapensis*, but not in the Spanish strain, HBS001 (4, 9).

### Nucleotide sequence accession number. 

The nucleotide sequence accession number is HF582854 (EMBL/GenBank).
